# Supplementary figures and images for: A fluorescence viewer for rapid molecular assay readout in space and low-resource terrestrial environments
Source: PLoS One. 2024 Mar 15;19(3):e0291158. doi: 10.1371/journal.pone.0291158 (PMC10942025; doi:10.1371/journal.pone.0291158)

not in paper

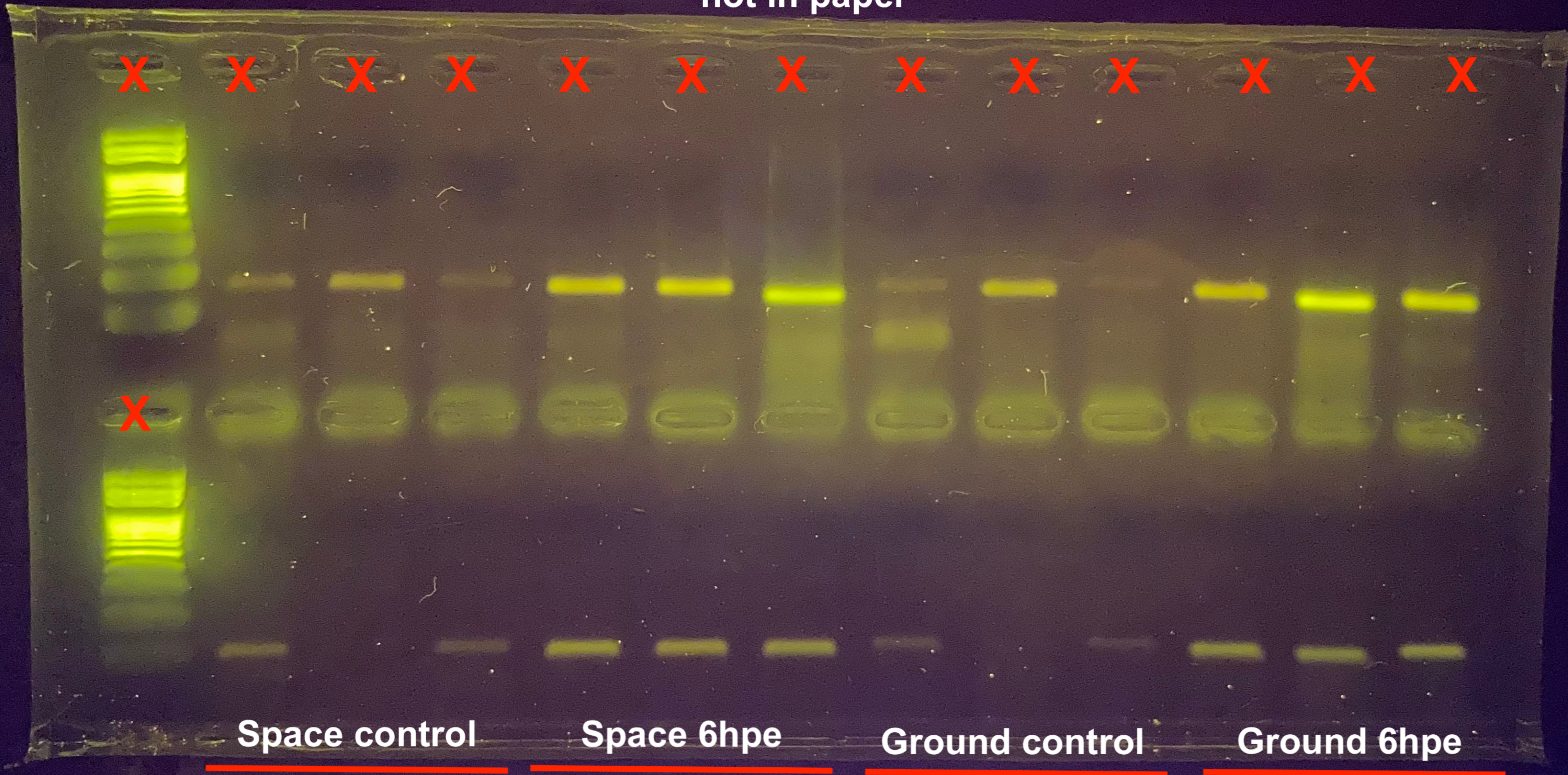

*cyp4a14* (Fig 4A)

Supplement: S1 Raw image — Sample order: 1–3. Space control. 4–6: Space 6hpe. 7–9: Ground control. 10–12: Ground 6hpe. (PDF) [file pone.0291158.s002.pdf]

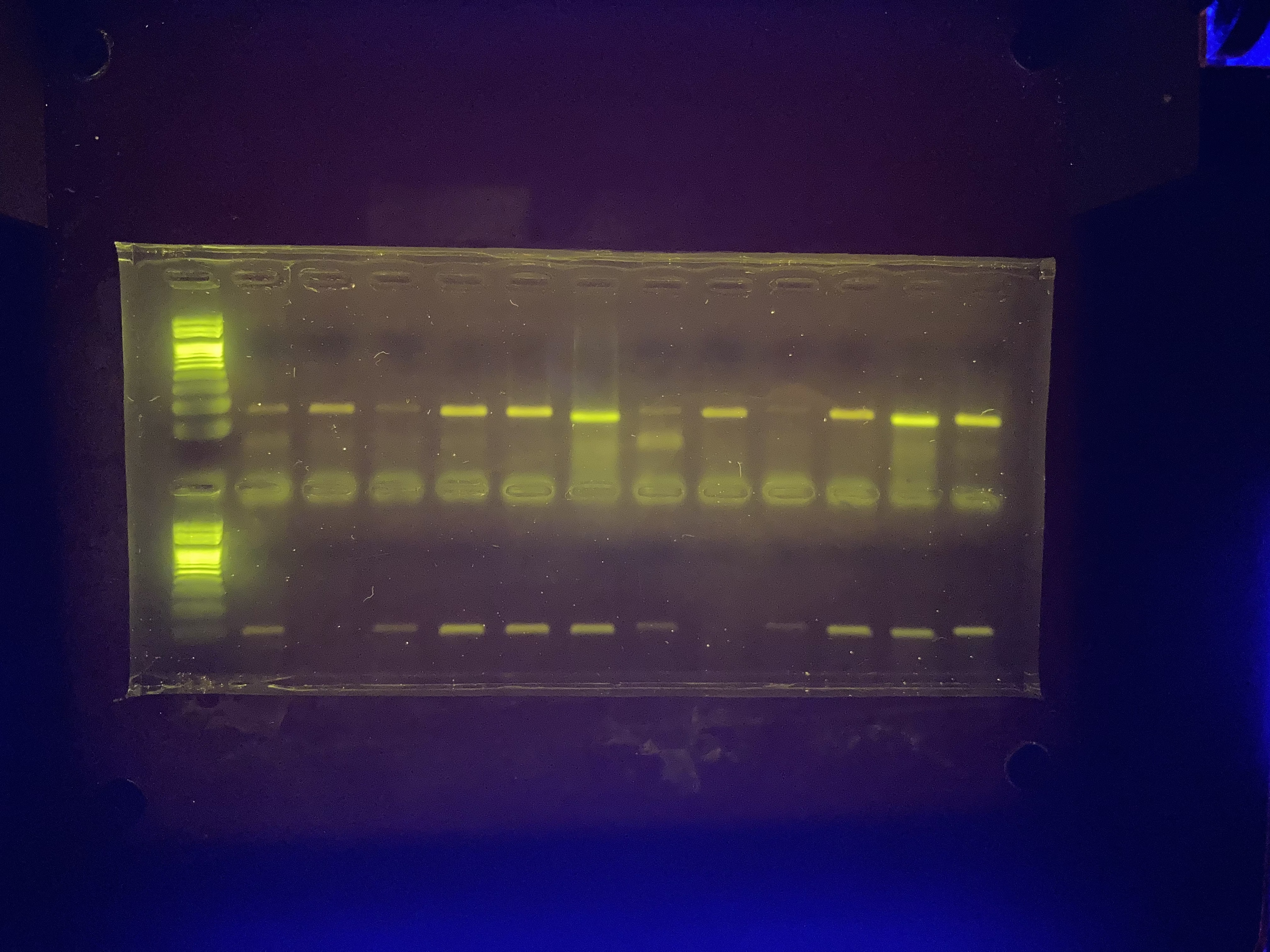

Supplement: S2 Raw image — Sample order: 1–3. Space control. 4–6: Space 6hpe. 7–9: Ground control. 10–12: Ground 6hpe. (TIFF) [file pone.0291158.s003.tiff]
