# Supplementary material for: A fluorescence viewer for rapid molecular assay readout in space and low-resource terrestrial environments
Source: PLoS One. 2024 Mar 15;19(3):e0291158. doi: 10.1371/journal.pone.0291158 (PMC10942025; doi:10.1371/journal.pone.0291158)
Supplement: Attachment — Submitted filename: Response to reviewers.docx [file pone.0291158.s005.docx]

**REVIEWER #1**

**1. Abstract (and methods, Figure 2): Author mentioned that heater is inside the PCR tube. Also, it seems so from the Figure 2. If that’s the case, how did you manage to control same temperature in all tubes at a same time? Moreover, what about risk of contamination? Reusability issue can be another factor. Please clarify.**

We thank the reviewer for highlighting several areas in the description of the GiS Viewer that could benefit from additional detail. We have updated the methods section to clarify the placement of the heating element within the GiS Viewer. The PCR tubes are placed within an aluminum block and a thin-film polyimide heater is adhered to the back of the aluminum block. Temperature regulation is then achieved through feedback from a thermistor located in proximity to the heater. The tubes remain closed throughout the incubation.

**2. Any specific Excitation/emission wavelength was used?**

We have updated the methods section to clarify that fluorophores are excited by blue LEDs with a spectral peak at 470 nm.

**3. In conclusion the authors mentioned about low power consumption. Please specify the power used for a PCR cycle or for the whole process.**

We have updated the methods to include maximum power data for both the GiS Viewer and the miniPCR.

**4. How did you manage to control of specific temperature of PI heater?**

We have updated the relevant methods sections to specify that temperature is controlled using a thermistor located in proximity to the heater in the GiS Viewer and a NTC thermistor with PID control in the miniPCR.

**5. What is the ramp rate during heating/cooling process?**

We have updated the methods to specify that the ramp rates in the miniPCR are 2.4 °C/s for heating and 1.7 °C/s for cooling.

**6. What element was used for cooling during PCR cycle?**

We have updated the methods to specify that fans are used for cooling in the miniPCR.

**REVIEWER #2**

**1. Analytical performance of the proposed method/device is not reported. It is important to clearly mention the basic analytical parameters such as LOD, sensitivity and measurement time.**

**If possible, compare these parameters with the other low-cost florescence readout systems. Also provide a tentative cost estimate of the device.**

We agree that these are important parameters. We have updated the text to specify the limit of detection for the RT-PCR and LAMP assays used here and the timeframes needed for detection. For the SARS-CoV-2 LAMP assay, we were able to detect 100 copies of viral RNA (lowest amount tested). For qPCR, we were able to detect a 3-4 fold difference in gene expression. Measurement time is assay dependent and we have updated the text to better clarify the imaging process. For assays that are continuously incubated at either room temperature, 72 °C, or 65 °C, imaging can be done in parallel with incubation and requires only turning on the LEDs and capturing an image using a phone or tablet. For assays that require incubation at varying or other temperatures, samples first have to be transferred into the GiS Viewer, which takes under a minute. We have also updated the text to specify the approximate manufacturing cost of the Viewer.

**2. Mention study limitations of the method in an appropriate place.**

We have added a section in the discussion detailing the major limitations of the study and the GiS Viewer.

**3. Figure 1A is difficult to follow. Please improve the figure quality; For example, a 3D schematic of the setup would help.**

We agree that Figure 1A would benefit from clarification. We have replaced the schematics with ones that better show the 3D structure of the GiS Viewer and the removable phone/tablet holder.

**4. Missing experimental details (Lines 14-25 page 10): Provide detail information of the excitation and collection filters, light source and heater used in the setup. Figure 1 is not cited in the lines 14-25 in page 10.**

We thank the reviewer for pointing out additional details needed in the methods section. These have now been added and Figure 1 has been cited as well.

**5) Introduction section needs improvements. With more references cited, make it easy to follow. For examples, several references can be cited in the first paragraph of introduction section.**

We have updated the introduction section to include additional citations to better situate the reader in the current context of the field.
